# The limits of global health diplomacy: Taiwan’s observer status at the world health assembly

**DOI:** 10.1186/s12992-014-0071-y

**Published:** 2014-10-01

**Authors:** Jonathan Herington, Kelley Lee

**Affiliations:** Department of Philosophy, Kansas State University, Manhattan, KS 66506 USA; Faculty of Health Sciences, Simon Fraser University, Blusson Hall, 8888 University Drive, Burnaby, BC V5A 1S6 Canada; Department of Global Health and Development, London School of Hygiene & Tropical Medicine, 15-17 Tavistock Place, London, WC1H 9SH UK

**Keywords:** Taiwan, World health organization, World health assembly, Global health diplomacy

## Abstract

In 2009, health authorities from Taiwan (under the name “Chinese Taipei”)^a^ formally attended the 62^nd^ World Health Assembly (WHA) of the World Health Organization as observers, marking the country’s participation for the first time since 1972. The long process of negotiating this breakthrough has been cited as an example of successful global health diplomacy. This paper analyses this negotiation process, drawing on government documents, formal representations from both sides of the Taiwan Strait, and key informant interviews. The actors and their motivations, along with the forums, practices and outcomes of the negotiation process, are detailed. While it is argued that non-traditional diplomatic action was important in establishing the case for Taiwan’s inclusion at the WHA, traditional concerns regarding Taiwanese sovereignty and diplomatic representation ultimately played a decisive role. The persistent influence of these traditional diplomatic questions illustrates the limits of global health diplomacy.

## Introduction

In 1972 the World Health Assembly (WHA) voted to formally recognise the People’s Republic of China (PRC), replacing Taiwan (Republic of China), as the legitimate representative of China [1]. This resolution, which aligned WHA membership with the rest of the United Nations (UN) system, was a by-product of broader shifts in the structure of East Asian international relations in the early 1970s [2]. Since this shift, there has been domestic and international disagreement over the terms upon which Taiwan’s population is represented at the UN, and this has had a profound effect on participation within technical agencies such as the World Health Organisation (WHO). In 1997, the Taiwanese government began to argue, on humanitarian and scientific grounds, that international health cooperation should not be subjugated to these broader political questions. Such arguments became especially salient as the 2002–2003 SARS (severe acute respiratory syndrome) outbreak, 2009 influenza pandemic, and other disease outbreaks of international concern, emphasized the trans-boundary nature of health risks in a globalising world.

In 2009, after twelve years of campaigning, a diplomatic breakthrough was achieved and Taiwan was invited to participate as an observer in the 62^nd^ WHA as “Chinese Taipei”. The agreement was widely hailed as a success for global health diplomacy, with the negotiations said to mark a new era of closer integration of health and foreign policy [3]. However, the apparent detente has since proven short-lived with a resumption of political disagreement in 2011 over the terms under which Taiwan could participate.

This paper analyses the process behind Taiwan’s efforts to gain observer status at the WHA between 2000 and 2011. The research draws on government documents, formal representations from both sides of the Taiwan Strait, and key informant interviews in Taiwan. The actors and their motivations, along with the forums, practices and outcomes of the negotiation process, are detailed. While it is argued that global health diplomacy was important in establishing the case for Taiwan’s inclusion at the WHA, traditional concerns regarding Chinese and Taiwanese sovereignty and diplomatic representation ultimately played a decisive role. The persistent influence of these traditional foreign policy questions illustrate the limits of global health diplomacy.

## Background

The WHA is the plenary decision-making body of the WHO, the United Nations specialised agency for health, and the main forum through which member states discuss and adopt resolutions pertaining to the organisation’s mandate. Along with member states, other entities can be granted permission to participate as non-voting observers at the WHA [4]. There are three formally recognised subtypes of observer status: (a) non-member states (currently only the Holy See); (b) representatives of the Palestinian and Occupied Territories under WHA Resolution 27.37; and (c) invited observers which participate at the annual invitation of the Director General (e.g. International Federation of Red Cross and Red Crescent Societies, Order of Malta, Inter-Parliamentary Union) [5,6]. Observer status has no legal standing under the WHO Constitution.

Taiwanese non-governmental interest in seeking observer status began in 1972 when medical professionals and related associations, particularly the Foundation of Medical Professionals Alliance in Taiwan (FMPAT), became concerned that the country’s exclusion would undermine domestic public health. At the diplomatic level, over the next twenty-five years, participation in WHO was not pursued as a specific foreign policy priority. Rather, the efforts of the Taiwanese government focused on regaining membership in the UN General Assembly as the sole representative of China [4]. It was not until 1997 that the Kuomintang (KMT)-led government began to specifically seek observer status at the WHA. Initial efforts came in the form of submissions, by WHO member states with diplomatic relations with Taiwan (such as Paraguay and Gambia), of a supplementary agenda item calling for the “Republic of China” to be admitted as an WHA observer. These proposals were typically rejected by the General Committee (responsible for finalising the WHA agenda) on the grounds that WHA Resolution 25.1 had determined in 1972 that the PRC is the sole representative of China within WHO (see, for instance, the record of debate on a proposal by Paraguay to invite “Taiwan” at the 2006 WHA [7]). These determinations implicitly endorsed the PRC’s “One-China” principle, whereby mainland China and the island of Taiwan (including other outlying islands) are considered to be parts of a single state, with a dispute between the “People’s Republic of China” and the “Republic of China” over who is the legitimate, internationally-recognised government of that state.

In 2000 the election of Democratic Progressive Party (DPP) leader Chen Shui-bian as president led to increased but ultimately unsuccessful efforts to gain observer status. It was not until the return to power of the KMT, under President Ma Ying-jeou, in 2008 that a breakthrough was achieved alongside improving cross-strait relations. In January 2009, the WHO Director-General, Margaret Chan, extended an invitation to the Taiwanese Centre for Disease Control (CDC) to act as a focal point under the 2005 revision of the International Health Regulations (IHR). This new status under the IHR, which is the WHO’s primary legal instrument for controlling the international spread of infectious disease, gave Taiwanese health officials the *in principle* opportunity to directly liaise with WHO officials, and attend WHO technical meetings. Finally, in April 2009, the WHO Secretariat invited the Taiwanese Department of Health to send a delegation of officials to attend the 62^nd^ WHA as observers under the name “Chinese Taipei”. Whilst Taiwan is a member of other international trade organizations (including the organization for Asia-Pacific Economic Cooperation and the World Trade Organization), the WHO is the first component of the UN system to grant formal status to Taiwanese representatives since 1972. Delegates of “Chinese Taipei”, though not able to vote as full members of the WHA, have been able to participate in floor debates, attend WHA side meetings, and formally lobby full members on issues of concern. This invitation process has since been repeated every year since 2009 [8].

Global health diplomacy (GHD) concerns “the multi-level negotiation processes that shape and manage the global policy environment for health” [9]. GHD has been described as the terrain where familiar forms of diplomacy are increasingly applied to health, as a nontraditional subject of diplomatic negotiations. Alcazar argues that this development is leading to a “revolution” or “paradigm shift” in foreign policy that could invert the hitherto low priority given to health [10].

To what extent can Taiwan’s observer status be hailed as exemplary of the role of global health diplomacy in international relations [11]? In particular, to what extent does this reflect a new development in diplomatic practice [12]. To better understand the nature of global health diplomacy, this paper analyses the negotiations surrounding Taiwan’s observer status in relation to five key questions:

1. What are the motivations of the parties involved?
2. Who are the actors involved in the process?
3. What forums did negotiations take place in?
4. What were the processes undertaken?, and
5. What were the outcomes of these processes.

By answering each of these research questions, this paper aims to evaluate the *impact* of health diplomatic actors, forums and processes, and thus determine whether a distinctive form of diplomacy is being practiced. The extent to which Taiwanese actors deployed a special form of diplomatic practice, and that this diplomatic practice mattered to the eventual resolution of the issue, is one of the central questions which this paper seeks to answer.

## Methods

A narrative and key-informant methodology was developed, focused on identifying the process, motives and actions of key Taiwanese actors during the period 2000 – 2008. Initial investigations and interview foci were developed through a multidisciplinary literature review of secondary sources, using a keyword search of PubMed and JSTOR, related to Taiwan, WHO and international health cooperation from the health sciences and international relations fields. Semi-structured interviews with nine key informants were then used to construct a narrative of the negotiation process leading up to the 62^nd^ WHA (including the actors, forums and processes) as well as the stated motivations of participants. Interviewees were identified with the assistance of the FMPAT in Taiwan (see Table 1 for demographics of interviewees), and included former members of the Taiwanese government under President Chen Shui-bian (2000–2008), non-governmental groups (FMPAT) and current Ministry of Foreign Affairs (MoFA) officials involved in the WHO observer negotiations. The sensitive nature of the Taiwan – PRC relationship posed a major challenge to this research project, as did continuing domestic political disputes between DPP and KMT actors over the nature of Taiwan’s relationship to the WHO. Only one official within the then serving Ma administration of the Kuomintang (KMT) government agreed to be interviewed. No official (current or former) from the PRC agreed to be interviewed for this research. To compensate for their under-representation amongst interviewee’s, we collected and analysed KMT and PRC official statements, WHO Summary Records, and media reports (from the English-language editions of the *China Post*, *Taiwan Times* and the PRC state news agency, *Xinhua*) to help provide insight into the process between 2008–2013.

**Table 1:** **Interviewee demographics**

**Affiliation**	**Approached**	**Interviewed**
NGO (Taiwan)	3	1
NGO (PRC)	1	-
Academic (Taiwan)	9	2
Academic (PRC)	2	-
Taiwan DoH (Former)	5	2
Taiwan DoH (Serving)	1	-
Member Of Parliament (Taiwan DPP)	4	1
Member Of Parliament (Taiwan KMT)	2	-
Taiwan MoFA (Former)	4	2
Taiwan MoFA (Serving)	2	1
PRC MoFA (Serving & Former)	1	-

The interviews were analysed to construct a narrative of negotiations during the study period. Key claims (about attendance, resolutions etc.) were cross-checked with media reports/official records where possible. The narrative was doubly verified via official documents, speeches, press releases and media reports within Taiwan and the PRC. Additional documents were obtained for WHO proceedings such as summary records of committee meetings, minutes of the WHA and Executive Board. These written sources were used to confirm and further develop the narrative of the negotiation process uncovered in the interviews.

### Ethics clearance

This research received ethics approval from the London School of Hygiene & Tropical Medicine Ethics Committee.

## Results

**Figure 1:**
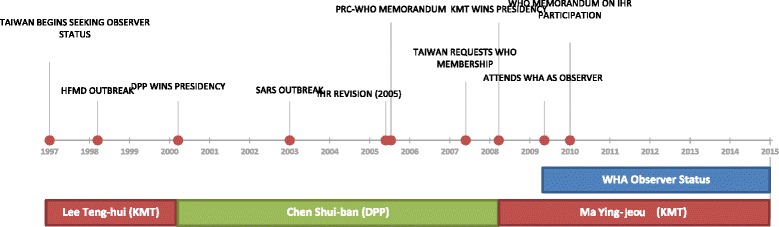
**Timeline of key events surrounding study period (2000–2011).**

The literature review and interviews were used to construct a timeline of the key events (Figure 1). Results are ordered according to the five key research questions.

### Motivations

Taiwan’s interest in formal participation in the WHA was motivated by both public health and foreign policy goals. As foreign policy, observer status was a potential means of leveraging participation in other international organisations and, in time, regaining recognition and legitimacy as a sovereign state. While this long-term aim enjoyed cross-party support, there were significant differences in approach between the KMT and DPP in relation to the WHA. The KMT, given what it termed “meaningful participation” and acceptance of the compromise label of “Chinese Taipei” [13], focused on securing a seat at the WHA as an end in itself. The DPP, as described by key informants, saw the achievement of observer status only as a step towards the longer-term goal of regaining Taiwan’s international standing [14]. Indeed, observer status was seen by many DPP interviewees as a regrettable compromise. In 2007, the DPP-led Taiwanese government submitted a request for full WHO membership, a significant departure from the previous goal of observer status [15].

The PRC’s longstanding opposition to Taiwan’s participation in the WHA stemmed from concerns that observer status would undermine Beijing’s claim that Taiwan is a province of the PRC governed by the Chinese Communist Party (CCP). It was this concern that led to the signing of a Memorandum of Understanding (MOU) between WHO and the PRC in 2005 which effectively limited WHO contact with Taiwanese officials to only those situations approved by Beijing [16]. The MOU coincided with negotiations to revise the International Health Regulations (IHR), concluded in 2005, and was seen as an attempt by the PRC to head-off any attempts to establish a direct link between the WHO Secretariat and an IHR national focal point in Taiwan. Instead of observer status, which Beijing saw as precedent setting, the PRC offered Taiwanese officials access to the WHA and WHO technical meetings as part of the PRC delegation [17].

### Actors and forums

The negotiation process for WHA observer status was driven (and resisted) by a number of different actors. In the main, Taiwanese interactions have involved far more diverse actors than their mainland counterparts who utilised (and benefited from) their status as the formally recognised diplomatic representatives of China (including Taiwan). Internally, the process of seeking observership was driven by the highest levels of the Taiwanese government, and supported by both the foreign policy and health ministries. Interviewees noted that the formulation of diplomatic strategy during the DPP period was directed from the Presidential office. Advice was sought from a panel of stakeholders within the government, including the Chairman of the Mainland Affairs Council (MAC), the Ministry of Foreign Affairs (MoFA), the Bureau of International Co-operation (BIC) within the Department of Health (DoH), and the Taiwanese Centre for Disease Control (CDC). The WHA issue was also elevated to the level of the National Security Council (NSC), a body normally concerned with matters of military defence. Key policy decisions, such as the 2007 decision to seek membership, were ultimately made by the President. These decisions were then promulgated through the various official and unofficial channels to link with a process of international lobbying of other governments and organisations for support.

Importantly, diplomatic actions by senior Taiwanese representatives remained severely constrained throughout. The PRC had placed a diplomatic ‘injunction’ against interactions with Taiwanese officials at the level of Vice-Minister or above [18]. Under Article 7 of the 2005 Memorandum of Understanding between the WHO Secretariat and China, for example, WHO agreed not to invite to the WHA or other meetings Taiwanese participants at the level of Director-General or above [15]. Much of Taiwan’s diplomacy at higher levels is thus conducted informally by proxies of the government. One interviewee, not formally engaged by the Taiwanese government, was specifically tasked with meeting a number of European ministers of health and foreign affairs to lobby for WHA participation. Others were involved in extensive lobbying of US congress members, resulting in the passing of the *Taiwan Participation Bill* and the formation of a vocal congressional caucus [19]. These informal avenues were seen as a viable way of circumventing PRC restrictions, and thus reassuring friendly governments, while still directly lobbying decision-makers.

Importantly, the strictures placed on Taiwanese diplomatic officials were less evident for health officials. Officials from the DoH and the CDC were able to interact relatively freely with their overseas counterparts, including the US Centers for Disease Control. Indeed, the Asia-Pacific Economic Cooperation (APEC) Health Taskforce (now the Health Working Group), where both Taiwan and the PRC are members, was initiated in part at the urging of Taiwanese health actors. Taiwanese interviewees familiar with the DoH recalled that this was one of the main conduits for information exchange between the Taiwanese authorities and their Beijing counterparts. Interactions with the WHO bureaucracy were, however, severely restricted. DoH and CDC officials with international expertise in specific public health issue areas had, up to 2005, been able to interact on a piecemeal basis as advisors to WHO, attending various technical meetings. In 2005, this arrangement was altered, under the conditions of the MOU, to a system where Taiwanese officials could only participate in a personal capacity after approval from Beijing’s MoH [15].

Given the above restrictions, one further set of actors in the WHA process was Taiwanese public health and medical professionals. Of particular importance, acknowledged by many interviewees, was the FMPAT which acts as an umbrella organisation for physicians and public health professionals in Taiwan. FMPAT actively and persistently lobbied international medical bodies, such as the World Medical Association (WMA) and the World Federation of Public Health Associations, to support Taiwan’s interaction with the WHO [20]. Interviewees claimed that the informal lobbying of health bureaucrats, scientific experts and health NGOs by Taiwanese health professionals kept the question of Taiwan’s status on the policy agenda. These informal civil-society interactions appear to have influenced other non-governmental actors, coinciding with an increasing focus on global health due to the 2003 SARS outbreaks and the 2008 change in government.

In the PRC, the Taiwan observer issue was predominantly dealt with by traditional diplomatic actors. Policy towards Taiwan is directed at the highest levels by the State Council which, in turn, is operationalised by a ministerial-level organisation, the Taiwan Affairs Office (TAO), and a ‘social organisation’, the Association for Relations Across the Taiwan Strait (ARATS). Ministry of Foreign Affairs (MoFA) officials led opposition to Taiwanese participation in the WHA, making formal representations to embassies in Geneva, the WHO Secretariat and on the floor of the WHA. The PRC was not, however, unaware of the proxy diplomatic influence wielded by Taiwanese NGOs and health professionals. Indeed, the PRC was concerned to limit their influence by seeking to “approve” the participation of Taiwanese nationals in NGO delegations. Some interviewees, with a public health background, noted that their participation at WHO meetings and at the WHA, as expert advisors or NGO delegates, was sometimes blocked, potentially at the behest of the PRC.

Bilateral interactions between the Chinese mainland and Taiwan, when they did occur, were mainly conducted between non-governmental and quasi-government organisations, as well as government officials in non-government capacities. Officially, Beijing recognises Taiwan as a province of China, meaning that official governmental interactions could occur between Mainland provincial officials (such as those from Fujian province) and Taiwan [21]. Cross-strait dialogue, mainly on economic and cultural issues, has traditionally taken place between ARATS and its Taiwanese counterpart, the Straits Exchange Foundation (SEF). Such contact between the PRC and Taiwan during the DPP period (2000–2008), however, became virtually non-existent owing to the mainland view that President Chen was a “stubborn Taiwan independence advocate” [22]. Relations with the KMT were more positive albeit controlled within certain parameters. Initiated by the 2005 meeting between Hu Jintao (General Secretary of the CCP) and Lien Chan (Party Chairman of the KMT) in 2005, contact between CCP and KMT officials occurred at the party level, although never in their capacity as formal state representatives. Further meetings since 2008 have seemingly solidified the relationship between the two parties [23].

### The process of global health diplomacy

The process of garnering support for Taiwan’s attendance at the WHA as an observer was complex. Several axes of negotiations were focused on the global health issues which Taiwan’s exclusion from the WHA exacerbated, while other arguments focused on traditional diplomatic concerns regarding sovereignty and the political relationship between Taipei and Beijing.

The decision to shift from WHO membership to WHA observer status, was viewed by many interviewees as a regrettable, but necessary, compromise. In contrast to the annual applications for UN membership, which were viewed as political symbolism, there was an added sense that WHA participation was both practically important and feasible. To this end, Taiwanese government officials, even those within the pro-independence DPP government, considered the pursuit of observer status (which does not imply statehood or sovereign legal status) a compromise position which the PRC could accept. Changes in the choice of moniker for the application process track the degree to which the traditional issue of sovereignty was entangled in the WHA process. Between 1997 and 2006, allied governments (normally Paraguay) introduced an agenda item inviting Taiwan to participate as an observer and initiating formal WHA discussion on Taiwan’s exclusion [14]. Taiwan initially sought invitation to the WHA under the name “Republic of China (Taiwan)” [24]. Increasingly less controversial names were used in applications between 2002 and 2006, including, ‘Health Authorities of Taiwan’ and ‘Taiwan, health entity’ [25]. Then, in the 2007 membership application, the deliberately provocative name of “Taiwan” was used. Some traditional allies who had previously supported observer status refused to support the 2007 bid, although a heated debate at the WHA was prompted when the agenda item was introduced by Taiwan’s diplomatic allies [14]. According to interviewees involved in the 2007 application, patience had worn thin. Although the repeatedly failed membership efforts were seen by some as a “fiasco”, after more than ten years of applying, there was a general sense of frustration with the continued refusal to grant WHA observer status. By 2007, the traditional issue of sovereign status and the WHA were once again conflated, with the WHA used as a forum for highlighting the ‘injustice’ of Taiwan’s exclusion from the international community.

Taiwan’s lack of formal recognition led to the use of two distinct diplomatic strategies. First, bilateral and informal lobbying of ‘friendly governments’, a long-established method of Taiwanese diplomacy, was once again deployed in the service of the WHA process. Interviewees, for instance, were involved in the lobbying of EU and US ministers and legislators see also [4]. The second strategy was use of the small cadre of countries who have diplomatic relations with Taiwan to push its case within official intergovernmental forums [26]. Although the two strategies relied on different actors and forums (one informal, one formal), they worked towards the same end: establishing the humanitarian and health case for Taiwan’s inclusion in the WHA. Interviewees noted that officials of friendly governments fully accepted the health case for Taiwan’s inclusion. Few governments, however, were willing to extend the argument within formal forums for Taiwan’s international recognition.

The cross-straits environment during the process of seeking WHA observership shifted multiple times. In broad terms, relations were closer from 2008 onwards than during 1997–2008. After eight years of pro-independence government, and increased cross-strait tensions, the return to power of the KMT in 2008 was accompanied by a milder diplomatic climate: what incoming President Ma Ying-jeou described as a “diplomatic truce” [27]. Two key points emerge. The first is that, for some time, the PRC’s position has been that dialogue was only possible with a Taiwanese government which accepted the 1992 consensus of a “One China, broadly interpreted” compromise. Under this consensus, both parties would agree on the ‘One China’ doctrine, but interpret differently *who* the rightful government of China is. The DPP, committed to full Taiwanese independence, has never supported the agreement [28]. The KMT has endorsed the 1992 consensus and, in its discussions with the CCP, reaffirmed this understanding as the basis of cross-strait co-operation [29]. Many interviewees with political ties to the DPP considered acceptance of the 1992 consensus by the KMT as a “major concession” to the PRC which damaged Taiwan’s status as an international entity [23].

### What outcomes were achieved by global health diplomacy?

On 28 April 2009 the Taiwanese DoH received a fax from the WHO Secretariat inviting “the Department of Health, Chinese Taipei” to send a delegation to the 62^nd^ WHA held the following month [30]. The letter, addressed to the Minister of Health Yeh Ching-chuan, ended 38 years of exclusion from the WHA. The Minister, Deputy Minister and senior health officials attended the WHA in 2009 and 2010 as observers under the name “Chinese Taipei” [31]. While lauded as a diplomatic breakthrough, in reflecting on the role of global health diplomacy, it is important to take account of a series of outcomes along the way. Moreover, the political arrangement eventually reached has proven to be more complex than first appeared.

The first outcome was the repeated failure to be invited as an observer to the WHA. Numerous procedural motions to place the issue of Taiwanese participation on the WHA agenda by its allies, notably Paraguay or Gambia, were regularly defeated [25]. The application for WHO membership in 2007 was also defeated. The second outcome was the 2005 Memorandum of Understanding between the WHO Secretariat and the PRC. The actual text of the MOU has not been publicly released, but an implementation document is available [15]. The memorandum significantly curtailed contact between the WHO Secretariat and Taiwanese authorities. As a result, Taiwanese representatives were able to attend only 21 of one thousand or so WHO technical meetings between 2005 and 2008 [32]. This practice reportedly continued in 2011 when Taiwan applied to take part in 21 WHO working panels and technical activities, of which eight were approved, nine were rejected and four received no response [33].

Third, the inclusion of a “universal application” clause in the revised IHR (2005), after lobbying by Taiwan and its allies, drew stark attention to Taiwan’s predicament. The inclusion of the clause allowed Taiwan and other “uncovered regions” to claim that they should be allowed to report and receive information through the IHR.

The final outcome was the eventual invitation to the WHA in 2009 and 2010. Although gaining observer status was greeted with equanimity by both the DPP and KMT, the terms by which Taiwan attended the WHA remain in dispute. According to the KMT, Taiwan participated in the two WHAs with almost the same rights and privileges as a full member state, serving as a model for participation in other intergovernmental bodies [34]. By DPP accounts, the terms of attendance reduced Taiwan to the same status as an international NGO [35]. The extent to which an annual invitation, granted yearly by the WHO Director-General, will continue in the future remains unclear. In 2011 and again in 2012, WHO informed the Taiwanese delegation that its named title would change, from “Chinese Taipei” to “Taiwan, province of China”. The decision led to immediate protest by Taiwan, as well as concern by the US Department of State at what seemed a unilateral decision by the WHO Secretariat [36].

## Discussion

The sensitivity of the PRC-Taiwan relationship resulted in KMT and PRC participants being under-represented amongst the key informants. We have attempted to control for potential data biases by interrogating the official statements of these under-represented parties, but it must be acknowledged that this limits, in particular, the scope of our insights into the motivations of these actors and the processes they undertook post 2008. Nonetheless, the sensitivity of both PRC and Taiwanese actors to the WHA issue should not merely be seen as a source of data bias, but also as an indicator of the continuing centrality of traditional political concerns. It is precisely the reticence of these actors to discuss the WHA process which suggests limits to the practice of “global health diplomacy” as a space where humanitarian concerns for health cooperation override traditional political concerns such as sovereignty. This is further reinforced by the swift change in the PRC’s attitude towards Taiwan’s WHA status, following the re-ascension of the KMT to power in 2008. It is reasonable to infer that the KMT’s long-standing commitment to the “One-China” policy, in contrast to the DPP’s professed desire for independence, were key in allaying PRC fears that WHA observer status would precipitate further formal diplomatic recognition elsewhere within the UN system.

Nonetheless, it would be a mistake to conclude that health concerns have no special status, and that attention to them rests solely upon traditional diplomatic processes, actors and forums. To begin with, it appears that health concerns have a special motivating force. It was the WHO’s plenary body alone which was targeted by both DPP and KMT governments for greater engagement whereby many UN bodies would have served the purpose of increasing diplomatic recognition. Two factors appear to explain the special status of health issues on Taiwan’s foreign policy agenda. First, the occurrence of two major public health events during this period intensified domestic political pressure to seek greater access to global health infrastructure. The first – an outbreak of hand, foot and mouth enterovirus (HFMD) in 1998 – affected a large number of schoolchildren and caused 78 deaths in Taiwan [37]. At the time, Taiwan was without a dedicated domestic disease outbreak investigation agency, and public health authorities also found themselves unable to access WHO assistance directly [38]. The outbreak led to the establishment of the CDC in Taiwan in 1999, and to increased public debate about the need for participation in WHO. The second event, the 2003 SARS outbreak, resulted in 680 cases and 81 deaths in Taiwan [39]. Once again, when cases were first identified in April 2003, the Taiwanese government attempted to notify WHO directly through official channels. However, without formal relations, official WHO notifications were delayed and direct technical assistance was even blocked [40]. While the outbreaks generated considerable public concern around Taiwan’s exclusion from the international health community, and intensified political pressure to resolve the diplomatic roadblocks, neither led to a change in the political impasse. Interviewees noted that, despite its institutional isolation, Taiwan dealt with the two events relatively well. Access to WHO’s technical expertise, although useful, was not considered essential by health officials.

Second, the international discourse around global health gave other member states and WHO itself, along with other global health institutions, a clear rationale for facilitating Taiwan’s participation. By 2009, the SARS outbreak was superceded by fears of an influenza pandemic, not only of the H1N1 virus that actually emerged, but the highly pathogenic H5N1 virus. Given Asia’s geographic proclivity to new outbreaks, the continued exclusion of a large population (23 million) from WHO activities was widely seen as problematic, especially amid the increasing transboundary mobility of health determinants and outcomes [32]. In short, global public health goals during this period may have intensified pressure on governments on both sides of the Taiwan Strait to reach agreement.

Third, it seems clear that novel diplomatic processes were critical to the positive case for Taiwan’s inclusion at the WHA, even as the presence of traditional sovereignty concerns continued to prevent resolution. Taiwan’s use of strategies characteristic of global health diplomacy – including bilateral lobbying of health officials, and the use of NGOs such as FMPAT and the WMA – were generally acknowledged by interviewees as crucial to keeping the issue at the forefront of WHA politics leading up to observer status. Although these strategies sometimes relied on traditional foreign policy actors and forums, they worked towards the same end - establishing the humanitarian and health case for Taiwan’s inclusion in the WHA. Without Taiwan’s status remaining a prominent fixture of official and unofficial WHA debates, it is unclear that the eventual détente in cross-strait relations would have yielded a change in WHA status.

Finally, whilst international and domestic pressure generated by Taiwan’s campaigning for observer status was influential, it seems clear that perennial concerns over the status of cross-Strait relations stymied progress. Both political parties in Taiwan acknowledge the importance of the 2008 detente but disagree over the extent to which it was the primary factor. The KMT government denied that the 2009 invitation was “approved” by China, although it is recognised that the invitation would not have been possible without improved relations between the CCP and the KMT [41]. Beijing, after eight years of dealing with a pro-independence DPP-led government in Taiwan, may have perceived the new KMT government as a more conducive partner for economic integration and a bulwark against Taiwanese nationalism [42]. Broader foreign policy concerns, namely an apparent desire to allay US government concerns about the tensions between the PRC and Taiwan, also gave the WHA negotiation process added impetus as a way of demonstrating improved relations [43]. Thus, there remains disagreement about the extent to which observer status was ‘bought’ with political concessions or whether it merely represented the fruits of a more cooperative cross-strait relationship.

## Conclusion

This paper has described Taiwan’s road to WHA observer status as a complex milieu of motivations, actors and forums. As well as helping to understand the potentially distinct features of global health diplomacy, this analysis illustrates its limits. First, the WHA process shows the increasing relevance of both state and non-state actors in diplomatic practice. Traditional diplomatic activities at the WHA were open only to the PRC and, whilst some governments which recognise Taiwan could act as proxies for its interests, most diplomatic channels remained closed to Taiwan. Both the 2000–2008 WHA campaigns, and the 2009 cross-strait détente, were conducted using a complex web of non-governmental and quasi-governmental entities – from both traditional political entities (e.g. KMT) and those focused on public health goals (e.g. FMPAT). While this suggests “new diplomacy”, at times these proxy actors were largely confined to interaction with their counterparts in health-specific forums or lobbying state representatives, rather than formal diplomatic engagement. Moreover, perhaps in recognition of their potential influence, the PRC then used its traditional diplomatic advantage to push for their exclusion from many WHO technical meetings. Thus, in contrast to the characterisation of new diplomacy as a process where bureaucrats form quasi-official networks to solve global governance problems [44], Taiwanese officials were only able to create a limited set of bilateral relationships and still sat outside some of the most important technical networks.

Second, the case seems to illustrate the continuing dominance of traditional foreign policy concerns, such as state sovereignty, over the priorities of global public health actors. The PRC’s overriding concern was to retain its longstanding foreign policy goal of ensuring that it is the sole recognised government of China (both the mainland and Taiwan). Once Taiwanese participation could be achieved on grounds that did not contradict, and perhaps even furthered this goal, Beijing relented. Nor were the motives of Taipei purely on health grounds. Although disease outbreaks generated domestic and international political pressure, the goal of accessing greater global health cooperation remained infused with foreign policy considerations. In particular, it is clear that participating as part of the Chinese delegation was politically unacceptable and there was thus a concomitant desire, particularly on the part of the DPP, to link observer status with full membership in WHO and ultimately the international community as a whole. In other words, foreign policy and health goals are potentially in competition with one another, and it is only when the latter does not create tension in the former that progress can be made.

Third, a sharp delineation between traditional and “new” diplomacy is difficult. Barred from formal diplomatic channels, the processes used by Taiwan to advance its cause demonstrated a canny understanding of these processes. The annual application by Taiwan’s allies to the WHA for observer status, for example, was critical to keeping the issue alive. Lobbying of governmental and nongovernmental counterparts worldwide by health professionals, officials and ministers was also continuous and well-organised. This diplomatic ‘guerrilla campaign’ contrasted with the formal diplomatic practice maintained by the PRC which focused on denying the separateness of Taiwan, and the reaffirmation of core UN principles of sovereignty and deference to the internal affairs of states. While complex and somewhat messy, traditional and new diplomacy came together to eventually produce agreement.

Finally, to what extent did global health diplomacy impact on the ultimate progression of events? Was the eventual breakthrough the result of the special contribution of global health diplomacy, or was it simply traditional diplomacy in action? Initially, it may be easy to discount much of the process of seeking observer status as meaningless. Without the change in Taiwan’s government in 2008, it is unlikely that cross-strait relations would have improved to the extent that the PRC felt willing to agree to Taiwan’s participation. Such a reductionist account, however, does not fully explain the fact that the WHA remains the only UN-affiliated organisation where Taiwan has an official presence. Other forums, such as the UN Framework Convention on Climate Change (UNFCCC) and International Civil Aviation Organization (ICAO), remain closed, suggesting that global health issues have a special status. Nor does the change in cross-strait relations explain why agreement was not reached in the 1990s, when relations were relatively good, following the agreement of the 1992 consensus, suggesting that the period of intensive global health diplomacy prior to 2008 was important in motivating moves towards Taiwan’s observer status at the WHA.

From the above analysis, we may conclude that, whilst global health diplomacy may have been important in making the positive case for Taiwan’s observer status, it was not sufficient alone to overcome longstanding foreign policy tensions. The question of Taiwan’s sovereignty, and the fluctuating temperature of cross-strait relations, needed to be addressed before health concerns could come to the fore. At the same time, global health diplomacy succeeded where other efforts to gain Taiwanese participation in the UN have failed. This suggests that health, as part of both old and new diplomacy, might occupy a potentially influential place in future global politics.

## Endnotes

^a^The use of ‘Taiwan’ in this paper should not be taken as recognition by the authors of a separate Taiwanese state, nor a rejection of the ‘One-China’ principle, but rather a simple appellation to describe the people and government who currently reside and possess control over the island of Taiwan and other outlying islands.
